# Mechanical Behavior of Elastic Self-Locking Nails for Intramedullary Fracture Fixation: A Numerical Analysis of Innovative Nail Designs

**DOI:** 10.3389/fbioe.2020.00557

**Published:** 2020-06-05

**Authors:** Giovanni Putame, Giulia Pascoletti, Mara Terzini, Elisabetta M. Zanetti, Alberto L. Audenino

**Affiliations:** ^1^Department of Mechanical and Aerospace Engineering, Politecnico di Torino, Turin, Italy; ^2^Polito^BIO^Med Lab, Politecnico di Torino, Turin, Italy; ^3^Department of Engineering, University of Perugia, Perugia, Italy

**Keywords:** intramedullary nails, Marchetti-Vicenzi nail, multibody analysis, flexible bodies, experimental tests, biomechanical stability, stiffness

## Abstract

Intramedullary nails constitute a viable alternative to extramedullary fixation devices; their use is growing in recent years, especially with reference to self-locking nails. Different designs are available, and it is not trivial to foresee the respective *in vivo* performances and to provide clinical indications in relation to the type of bone and fracture. In this work a numerical methodology was set up and validated in order to compare the mechanical behavior of two new nailing device concepts with one already used in clinic. In detail, three different nails were studied: (1) the Marchetti-Vicenzi's nail (MV_1_), (2) a revised concept of this device (MV_2_), and (3) a new Terzini-Putame's nail (TP) concept. Firstly, the mechanical behavior of the MV_1_ device was assessed through experimental loading tests employing a 3D-printed component aimed at reproducing the bone geometry inside which the device is implanted. In the next step, the respective numerical model was created, based on a multibody approach including flexible parts, and this model was validated against the previously obtained experimental results. Finally, numerical models of the MV_2_ and TP concepts were implemented and compared with the MV_1_ nail, focusing the attention on the response of all devices to compression, tension, bending, and torsion. A stability index (SI) was defined to quantify the mechanical stability provided to the nail-bone assembly by the elastic self-locking mechanism for the various loading conditions. In addition, results in terms of nail-bone assembly stiffness, computed from force/moment vs. displacement/rotation curves, were presented and discussed. Findings revealed that numerical models were able to provide good estimates of load vs. displacement curves. The TP nail concept proved to be able to generate a significantly higher SI (27 N for MV_1_ vs. 380 N for TP) and a greater stiffening action (up to a stiffness difference for bending load that ranges from 370 Nmm/° for MV_1_ to 1,532 Nmm/° for TP) than the other two devices which showed similar performances. On the whole, a demonstration was given of information which can be obtained from numerical simulations of expandable fixation devices.

## Introduction

Intramedullary fixation devices have gained popularity in recent years thanks to the improvement in nail design, especially with reference to axial and rotational stability of the fracture. Compared to extramedullary devices (e.g., plates for osteosynthesis), intramedullary fixation has the biomechanical advantage of optimizing load sharing between bone and the device itself; with reference to surgery, they are less invasive and more likely to preserve the periosteal blood supply (Modabber and Jupiter, 1998). The current indication of intramedullary fixation devices is for specific fractures (e.g., unstable intertrochanteric fracture; Bonnaire et al., 2012), while their superiority compared to extramedullary devices is still the object of many debates, with some positive confirms (Rehman et al., 2015; Yu et al., 2018) as well as some contraindications related to later bone fracture risk (Parker and Handoll, 2010), leading to the generic assumption that extramedullary fixation devices are mostly indicated for unstable fractures (Schipper et al., 2004).

Intramedullary nails with different constructions and working principles were proposed. In general, they can be distinguished between inter-locking and self-locking nails. Contrary to inter-locking nails, which are constrained to the bone segments through proximal and distal fixation screws, self-locking nails remove the need of distal inter-locking screws through the adoption of alternative anchoring mechanisms. Specifically, such mechanisms imply the expansion of the device which gets in contact with the inner surface of the medullary canal, thus providing the distal locking. Among the used expansion mechanisms deserving to be mentioned there are: the releasing, by means of a sliding nut, of the distal ends of a bundle of curved elastic rods which constitute the nail body (see the Marchetti-Vicenzi nail, Anastopoulos et al., 2001); the spreading out of a series of anchoring flanges, controlled by the tightening of a bolt located at the distal end of the nail (see the Seidel nail, Giudice et al., 2006); the expansion of the whole nail body, consisting of a folded stainless steel shell, through the injection of a pressurized saline solution (see the Fixion nail, CarboFix Orthopedics Ltd., Herzliya, Israel). A few works dedicated to study these nails are reported in literature, most of them refer to Marchetti-Vicenzi nail (Tennant et al., 2001; Madan et al., 2003; Martínez et al., 2004) or other fixation systems (Rose et al., 2013). These works report that, thanks to their working principles, expandable self-locking nails should offer some main advantages such as: a reduced surgical invasiveness due to the possibility of avoiding reaming procedure, thus ensuring shorter operative time; lower blood loss and a reduced exposure to X-ray thanks to the absence of a distal inter-locking screws implantation (Zhou et al., 2015); minimized risk of mechanical failure as a consequence of stress concentration at the nail-screw interface (Bath et al., 2006). Furthermore, the bone near fracture rim is not bypassed by the nail (as it is with inter-locking nails prior to dynamization) and therefore bone remodeling is promoted.

Although clinical studies have highlighted the advantages of expandable nails over inter-locking nails, to date, conflicting results are reported in the literature, especially with regard to the treatment of lower limb fractures (Hargreaves et al., 1996; Simon et al., 1997; Smith et al., 2006; Kapoor et al., 2009). For instance, Hargreaves et al. (1996) observed mal-alignment, non-union and shortening of femoral and tibial fractures in patients treated with the Marchetti-Vicenzi nail. Conversely, for the same device, Simon et al. (1997) reported satisfactory clinical outcomes for the treatment of femoral shaft fractures. Furthermore, from the biomechanical point of view, several works demonstrated that these devices might not ensure adequate torsional and axial stability (Ivanov et al., 2016). As a consequence, traditional inter-locking nails still represent the first choice in the clinical practice. However, in light of the significant benefits provided by expandable nails, it is worth looking into new strategies to improve the respective biomechanical performance.

Clinical trials and meta-analyses are the main tool to compare the performances of different devices. Nonetheless, these studies are affected by some major shortcomings: they may fail to reach consistency due to heterogeneity in treatment groups as a result of the variability of surgeon's experience (it could be significantly shorter for the most recent devices), to different indications in relation to fracture complexity and to patient activity level. In addition, in most cases there was no blinding of assessors or patients (Rose et al., 2013). It is therefore highly desirable being able to establish some performance indices prior to clinical trials.

The present research work aims to give a contribution in this direction, providing a numerical tool for the pre-surgical screening of internal fixation devices. Working *in silico* provides some main advantages (Terzini et al., 2017; Zanetti et al., 2017; Putame et al., 2019a,b): it avoids burden on living subjects and, secondly, the exact same conditions can be replicated, isolating the effects produced by the fixation device alone. Three elastic self-locking intramedullary nails are here examined as a benchmark, including a Marchetti-Vicenzi nail and two new nail concepts. Furthermore, specific indices to be used to compare the respective stability and stiffening action are proposed.

## Materials and Methods

### Expandable Nail Designs

**Figure 1:**
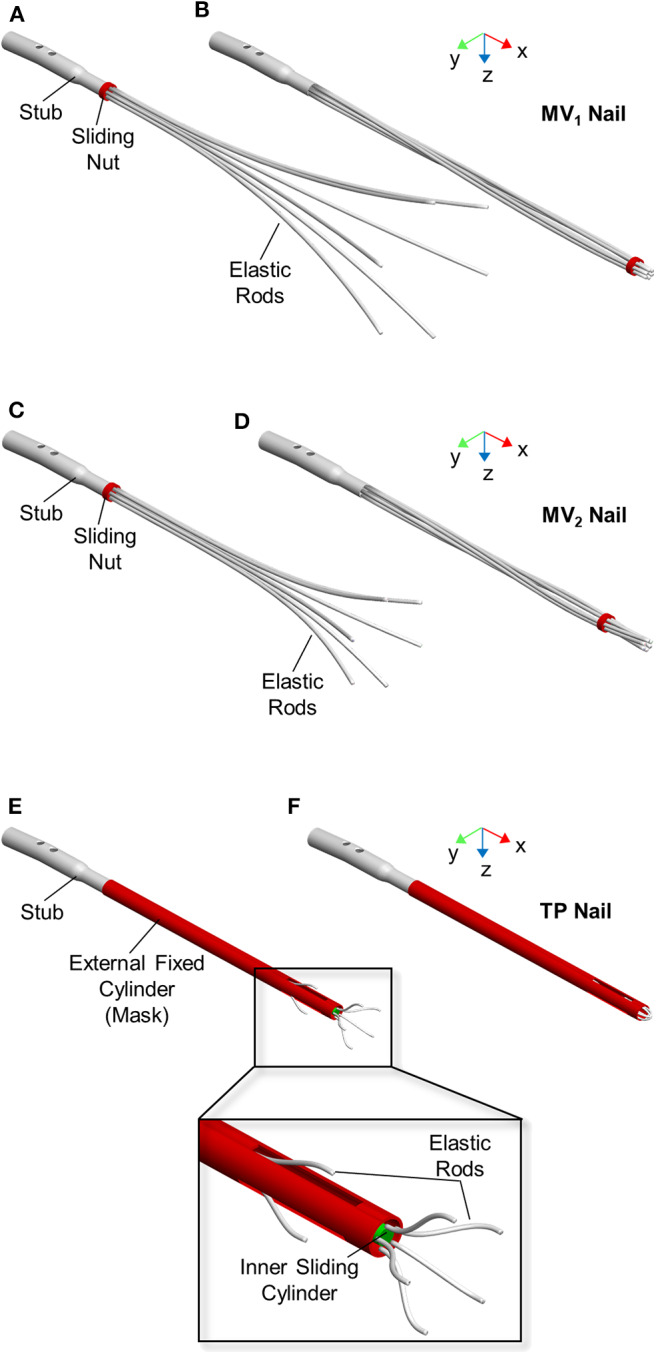
Expandable nail designs. From top to bottom: MV_1_ nail, MV_2_ nail, and TP nail. Devices are showed in open **(A,C,E)** and closed **(B,D,F)** configuration.

Three different elastic self-locking nails were analyzed, including a physical device and two new numerically modeled designs. The first nailing device is the latest developed version of the elastic nail designed by Marchetti-Vicenzi (Anastopoulos et al., 2001; Zanetti et al., 2008), which will be called MV_1_ (Figures 1A,B) in the following. It is made of six elastic rods (circular cross-section with diameter of 3 mm) which are kept closed by a sliding nut in order to allow their simultaneous introduction into the medullary canal. After the implantation, the sliding nut is withdrawn so that the elastic rods are free to elastically expand into the medullary cavity and stabilize the fracture.

**Figure 2:**
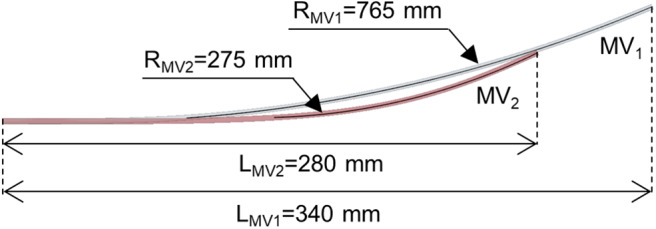
Elastic rod geometry of the MV_1_ nail (gray rod) and MV_2_ nail (red rod) models. Length (L) and bending radius (R) are indicated. The rod diameter is equal to 3 mm for both models.

The second nail design concept (MV_2_) is a revised version of MV_1_ (Figures 1C,D). Here the six elastic rods (circular cross-section with a diameter equal to 3 mm) are shorter (L_MV2_ = 280 mm) and with a smaller bend radius (R_MV2_ = 276 mm) compared to MV_1_ (L_MV1_ = 340 mm, R_MV1_ = 765) (Figure 2).

The third nail design concept, called “Terzini-Putame” nail (TP), presents two groups of radially equidistant elastic rods (circular cross-section with a diameter equal to 1.5 mm) departing from two different axial positions along a sliding inner cylinder (Figures 1E,F). In particular, the device is composed of 3 proximal rods and 5 distal rods. An external hollow cylinder, fixed to the nail stub, characterized by the presence of three radially equidistant slots, acts like a mask keeping the elastic rods closed as long as the inner cylinder slides distally letting them open.

### Numerical Models

A multibody approach was chosen since many parts can be assumed as solid bodies (i.e., the bones fragments and nail components). The only exception were the elastic rods coming into contact with the medullary canal, which were modeled as flexible bodies by means of a software-native tool, included in ADAMS View (2017, MSC Software, Santa Ana, CA), able to manage large deformations in beam-like structures. This tool allowed to create each flexible rod as a beam made of consecutive Finite Elements (so called FE Parts, MSC SimCompanion, 2016a), having specified the rod centreline, cross-sectional properties (i.e., area and moments of inertia derived from the rod diameter), material properties, and a series of nodes that determine the number of discretization elements. In detail, the rods were defined by 10, 7, 8, and 11 nodes for the MV_1_, MV_2_, TP proximal, and distal rods, respectively. The constituting material of all devices was stainless steel AISI 316 LVM with a density equal to 8,000 kg/m^3^. Concerning flexible rods, the material was assumed to be isotropic and perfectly elastic with a Young's modulus equal to 187.5 GPa and a Poisson's ratio equal to 0.33. The 3D geometry of the femoral cortical bone was derived from a Sawbones left femur model (SKU: 3908). Thereafter, a diaphyseal fracture was reproduced, thus obtaining two bone segments (i.e., proximal and distal segment). A density of 2,000 kg/m^3^ was assigned to the bone.

#### Boundary Conditions

Simulations of each device behavior required performing three consecutive steps: (1) nail closure and its implantation inside the medullary canal, (2) nail opening inside the medullary canal, and finally, (3) loading of the bone-nail system.

With reference to MV_1_ and MV_2_ models, device closure and its successive opening were realized fully constraining the proximal end of the elastic rods to the stub and applying a motion to the sliding nut in order to let it translate along the nail axis (x axis in Figure 1), while it could freely rotate around the same axis. Concerning the TP model, the proximal end of the elastic rods was fully constrained to the inner cylinder. A motion was applied to the sliding cylinder in order to let it translate along the nail axis (x axis in Figure 1).

After the nail opening step, three different loads were applied on the bone-fixation device systems:

– Axial loads (both tension and compression)
– Torsional loads (in both directions)
– Bending loads (according to the four-point bending configuration)

**Figure 3:**
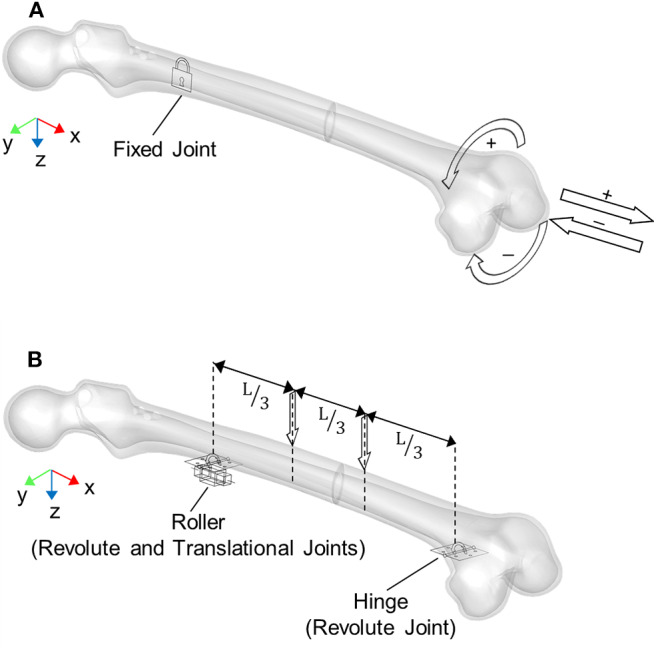
Boundary conditions applied for loading simulations: **(A)** axial and torsional configurations, **(B)** four-point bending configuration where L is the distance between the roller and hinge constraints. Arrows represent applied loads directions.

The axial loads were applied on the distal end of the femur and the loading direction was coincident with the expandable nail axis (Figure 3A). Tension and compression simulations were implemented through the application of an axial force ramping up to 100 N. In addition, axial displacements were limited to 50 and 15 mm for tensile and compression loads, respectively, in order to early stop the simulations. These limits were set since such high displacements were assumed not to be compatible with an *in vivo* realistic fracture progression.

The torsional loads were applied on the distal end of the femur as well, with a moment axis coincident with the expandable nail axis (Figure 3A). In detail, for both positive (internal femur rotation) and negative (external femur rotation) torsion, the torque was applied following a ramp from 0 to 100 Nmm. In this case, a rotation limit equal to 90° was set.

The four-point bending was realized as depicted in Figure 3B: a hinge and a roller were placed below the femur at the distal and proximal fragment, respectively. Two downward forces, ranging from 0 to 100 N, were then applied on the upper femur surface, with an inner axial distance L = 252 mm.

#### Contact Parameters

All contact forces (between elastic rods and bone and between nails and the sliding stub/cylinder) were simulated through the “IMPACT” formulation in ADAMS (MSC SimCompanion, 2016b). According to this contact formulation, the force is computed as a function of both the interpenetration depth and the relative velocity between colliding bodies. For MV_1_ and MV_2_ devices, contact forces were defined between the elastic rods and the distal and proximal femur segments, and among all elastic rods; with reference to TP model, contacts were defined only between the elastic rods and the distal bone fragment (due to the device geometry) and between the external cylinder and the whole bone. No contacts were defined between the fracture surfaces in order to investigate and compare the biomechanical performance of devices regardless of the specific fracture typology. For contacts involving the bone, an additional friction force component was considered, exploiting the following parameters retrieved from literature (Parekh et al., 2013):

– Static coefficient μ_s_: 0.59
– Dynamic coefficient μ_d_: 0.59
– Stiction transition velocity v_s_: 10 mm/s
– Friction transition velocity v_d_: 100 mm/s

### Stability Assessment

Two indices were set up to assess expandable nails performances. The first index is the stability index (SI), able to quantify the mechanical stability provided by the elastic nail expansion against relative movements between the device and the bone. It was calculated as the sum of contact forces between the elastic rod and the bone at the end of device opening inside the medullary canal:

(1) $$\begin{array}{r} SI=\overset{N}{\underset{i=1}{\sum }}F_{ni} \end{array}$$

where:

– *N* is the total number of elastic rods
– *F*_*ni*_ is the contact force between the *ith* nail and the bone

*SI* would represent the friction force (*F*_*t*_) between the two components (i.e., the bone and the nail) for a friction coefficient equal to one and all-parallel friction forces:

(2) $$\begin{array}{r} F_{t}=\overset{N}{\underset{i=1}{\sum }}F_{ti}=f\;\overset{N}{\underset{i=1}{\sum }}F_{ni}=f\cdot SI \end{array}$$

where

– *f* is the static friction coefficient between each elastic rod and the bone
– *F*_*ti*_ is the friction force between the *ith* elastic rod and the bone

The second index is represented by the stiffness of the bone-nail system for different external loads: tension, compression, torsion and bending. In detail, secant (*k*_*S*_) and tangent (*k*_*T*_) stiffnesses were derived from reference points on the force/moment vs. displacement/rotation curves, as described in the following.

The secant axial stiffnesses of the bone-device system for tension and compression simulations were evaluated at the displacement limits of 50 mm (*k*_*S*__50 mm_) and 15 mm (*k*_*S*__15 mm_), respectively, as the ratio between the applied force and the bone fragment displacement. The tangent axial stiffnesses were computed as the slope of the tangent line where the force starts rising (*k*_*T in*_): given the variability of the traction and compression curves, the points at which this tangency was assessed were defined a posteriori on the basis of obtained results. For the compression load, an additional tangent stiffness was considered: the slope of the tangent line at a translational displacement equal to 0.1 mm (*k*_*T*__0.1mm_).

The secant torsional stiffnesses of the bone-device system was evaluated at 50 and 100 Nmm (maximum applied torque) as the ratio between the applied moment and the respective bone fragment rotation (*k*_*S*__50 Nmm_ and *k*_*S*__100 Nmm_). The tangent torsional stiffness was calculated as the slope of the tangent line at 50 Nmm torque (*k*_*T*__50Nmm_).

The secant bending stiffnesses were calculated at 900 Nmm (*k*_*S*__900 Nmm_) and 8,400 Nmm (*k*_*S*__8400 Nmm_) according to Equation (3):

(3) $$\begin{array}{r} k_{s}=\frac{M}{\theta } \end{array}$$

where:

*M*: bending moment (900 or 8,400 Nmm)

θ: bending angle (sum of the distal and proximal fragments rotations)

In detail, the bending moment and the respective bending angle were computed according to Equations (4) and (5).

(4) $$\begin{array}{r} M=F\cdot \frac{L}{3} \end{array}$$

(5) $$\begin{array}{r} \theta =|\theta_{d}|+|\theta_{p}| \end{array}$$

where:

*F*: external vertical force

*L*: distance between joints (Figure 3B)

θ_*d*_: distal fragment rotation

θ_*p*_: proximal fragment rotation

The tangent bending stiffness was computed as the slope of the tangent line at 900 Nmm bending moment (*k*_*T*__900Nmm_).

### Model Validation

**Figure 4:**
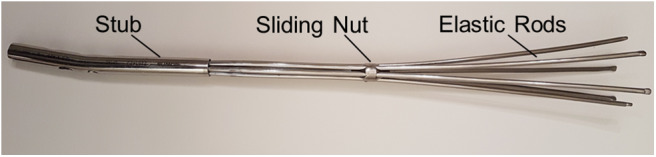
MV_1_ physical device used during the experimental tests.

**Figure 5:**
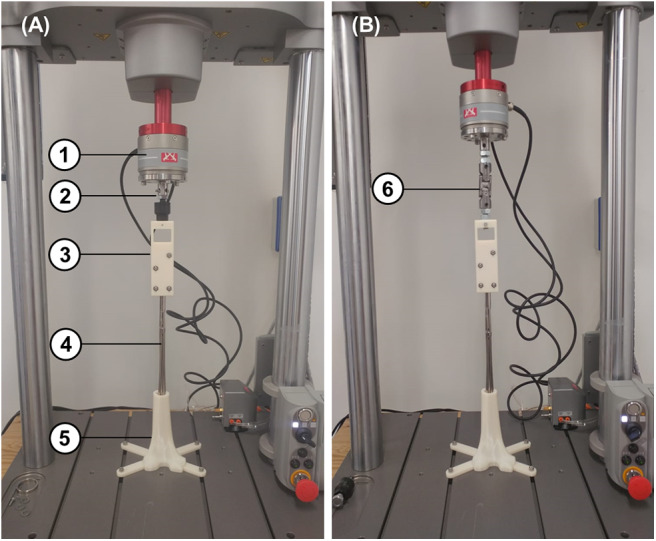
Experimental tensile **(A)** and torsional **(B)** test set up consisting of: (1) load cell, (2) spherical joint for tensile tests, (3) stub clamp, (4) MV_1_ device, (5) 3D printed distal femur, and (6) double cardan joint for torsional tests.

All numerical models share the same modeling approach, therefore only the MV_1_ model was experimentally validated, since this is the only device that was physically available at the moment (Figure 4). Two different experimental set ups were designed (Figures 5A,B), for translational and rotational tests. In both set ups the distal part of the fractured femur was manufactured in ABS plus-P430 thermoplastic printing material (Stratasys, Eden Prairie, Minnesota, USA), according to the respective CAD model (STL file), through additive manufacturing (uPrint SE Plus, Stratasys). The printed bone segment was screwed to the lower fixture of a testing machine (Instron E3000) by means of protrusions added externally to the bone geometry. The nail was inserted, in its closed configuration, into the printed distal femur fragment in order to match the insertion depth of the respective numerical model, which was equal to about 165 mm when measured as the distance between the fracture surface and the nail tip. Successively, the sliding nut was manually withdrawn to allow the nail expansion within the synthetic bone. All tests were controlled by displacement signal and the respective force was measured by the machine load cell (with a range equal to ±5 kN for linear loads and to ±25 Nm for rotational loads). As shown in Figure 5, a spherical joint and a double cardan joint were alternatively used to connect the test machine to the tested device during tensile and torsional tests, respectively. In detail, a translational velocity of 1 mm/s was imposed for tensile tests with a displacement limit equal to 50 mm, while an angular speed of 2°/s was set for torsional tests together with rotation limits equal to −90° and 90°.

## Results

### Numerical and Experimental Comparison

MV_1_ numerical model was validated against experimental results (as described in the “Model Validation” section). Numerical simulations with applied translational displacements and positive or negative rotations were performed, reproducing the experimental loading conditions.

Preliminary numerical results have shown that the tensile force is almost constant for different translational displacements and it is extremely low (about 6 N for displacements ranging from 0 to 50 mm). In addition, the system response is biased by the initial action of the elastic rods which tend to move the bone, due to their curvature. For these reasons experimental test results from tensile loads were only used for a qualitative validation. On the contrary, torsion tests have produced higher loads, making this type of test optimal for performing a quantitative validation.

**Figure 6:**
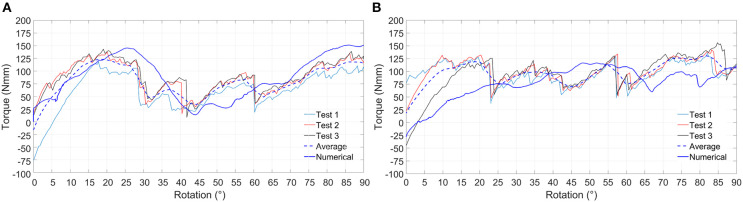
Experimental and numerical results relative to positive **(A)** and negative **(B)** torsion loading condition. Experimental curves (Test 1, Test 2, and Test 3), average experimental curve and numerical curve are compared.

In Figure 6 results for the positive and negative torsion tests are shown. As it can be seen, the numerical results closely follow the average experimental curve for both positive and negative torsion loads.

### Stability Assessment

**Figure 7:**
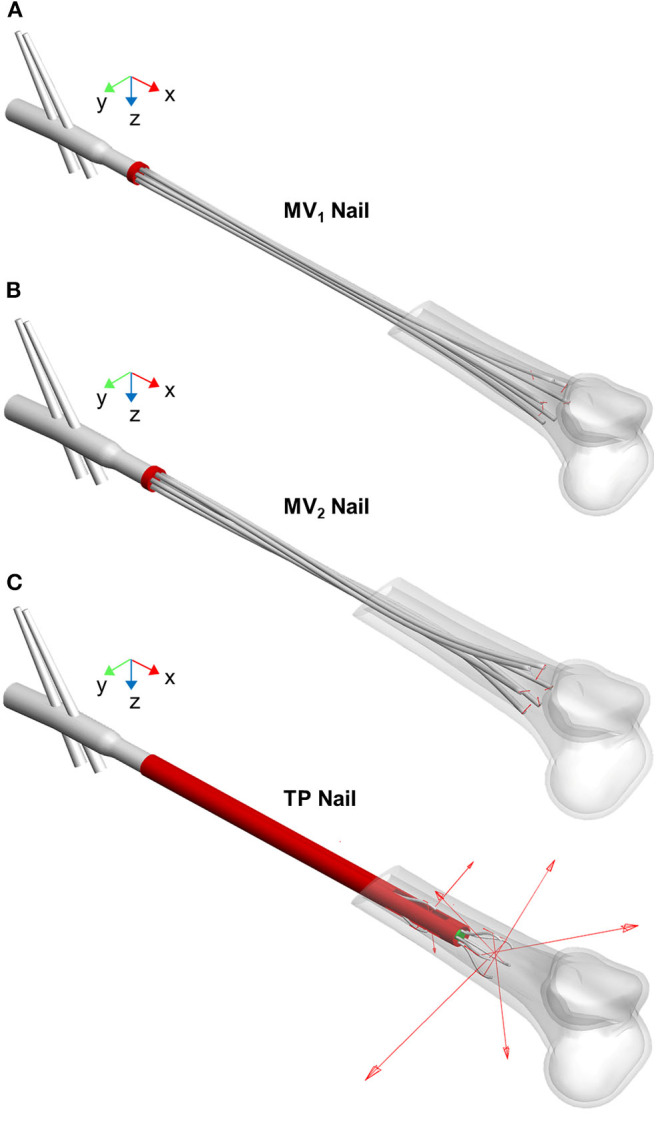
MV_1_ nail **(A)**, MV_2_ nail **(B)**, and TP nail **(C)** at the end of the opening step simulation (the proximal femur is not shown). The elastic rods positioning after the implant procedure is visible as well as the vector contact forces (red arrows) exchanged between rods and medullary canal.

Figure 7 shows all the implanted nails at the end of the simulated expansion step. Devices performance in terms of stability was evaluated through the stability index (SI) and the stiffness parameters.

In Table 1 the stability index values for the three devices are listed. The TP device shows the best performance with reference to this index; indeed, the sum of the normal components of all nails contact forces assumes the highest value compared with MV_1_ and MV_2_ models. This result implies that, at the end of the opening phase, the relative motion between the elastic rods and the medullary canal requires the highest tangential force for the TP device. On the other side, MV_1_ and MV_2_ devices show comparable performances.

**Table 1:** Stability Index (SI) for the three devices.

	**MV_**1**_**	**MV_**2**_**	**TP**
SI [*N*]	27.27	33.61	380.02

**Figure 8:**
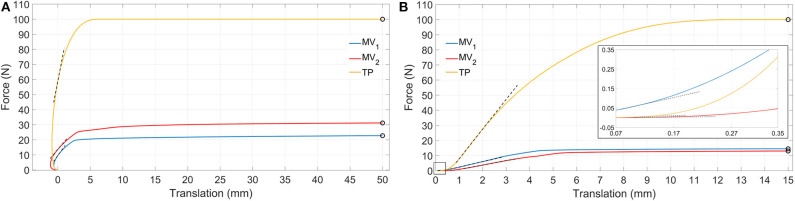
Simulation results showing tension **(A)** and compression **(B)** force vs. translation. Dashed black lines represent tangent lines used for the evaluation of *k*_*T in*_ and *k*_*T*__0.1 mm_ (box in **B**) parameters, while circle markers identify reference points used for *k*_*S*__50 mm_ and *k*_*S*__15 mm_ parameters computation (for the sake of clarity secant lines are not reported in figure).

**Figure 9:**
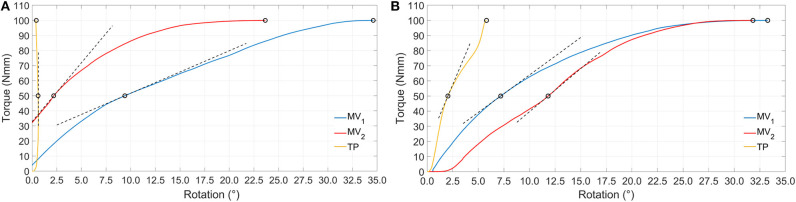
Simulation results showing positive **(A)** and negative **(B)** torque vs. rotation. Dashed black lines represent tangent lines used for the evaluation of *k*_*T*__50 Nmm_ parameter, while circle markers identify reference points used for *k*_*S*__50 Nmm_ and *k*_*S*__100 Nmm_ parameters computation (for the sake of clarity secant lines are not reported in figure).

Moving to stiffness evaluation, as previously described, several stiffness measures were computed in the explored loading conditions. Results of tension/compression simulations are reported in Figure 8 and Table 2 lists the resulting axial stiffnesses. Positive and negative torsional stiffnesses are listed in Table 3 while Figure 9 shows the torsional trends. Both in relation to tension/compression and torsional results, TP stiffness is considerably higher than MV_1_ and MV_2_ (up to 79 times higher for positive torsion simulations). Conversely, MV_1_ and MV_2_ show similar behavior, but MV_2_ outperforms the original Marchetti-Vicenzi design in the positive torsion condition, reaching a stiffness up to 33 times higher than MV_1_.

**Table 2:** Tensile and compression stiffness parameters.

	**MV_**1**_**	**MV_**2**_**	**TP**
**TENSION SIMULATIONS**			
*k*_*T in*_ [N/mm]	6.00	5.12	22.17
*k*_*S* 50 mm_ [N/mm]	0.46	0.62	2.00
**COMPRESSION SIMULATIONS**			
*k*_*T* 0.1 mm_ [N/mm]	0.67	0.03	0.11
*k*_*T in*_ [N/mm]	3.57	2.93	19.67
*k*_*S* 15 mm_ [N/mm]	0.97	0.87	6.67

**Table 3:** Positive and negative torsion stiffness parameters.

	**MV_**1**_**	**MV_**2**_**	**TP**
**POSITIVE TORSION SIMULATIONS**			
*k*_*T* 50 Nmm_ [Nmm/°]	2.82	7.80	∞
*k*_*S* 50 Nmm_[Nmm/°]	5.31	22.83	81.97
*k*_*S* 100 Nmm_[Nmm/°]	2.89	4.23	232.56
**NEGATIVE TORSION SIMULATIONS**			
*k*_*T* 50 Nmm_ [Nmm/°]	4.95	5.69	16.07
*k*_*S* 50 Nmm_ [Nmm/°]	6.95	4.23	24.63
*k*_*S* 100 Nmm_ [Nmm/°]	3.01	3.14	17.24

**Figure 10:**
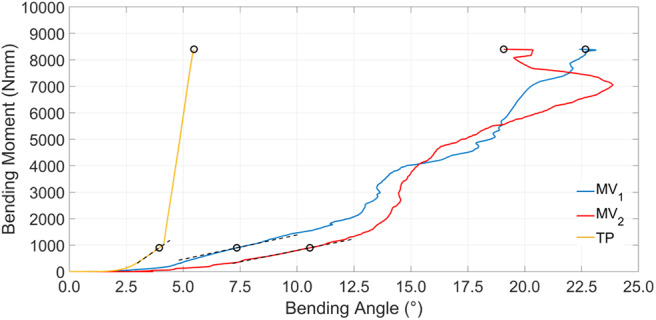
Simulation results showing bending moment vs. bending angle. Dashed black lines represent tangent lines used for the *k*_*T*__900 Nmm_ parameter evaluation, while circle markers identify reference points used for *k*_*S*__900 Nmm_ and *k*_*S*__8400 Nmm_ parameters computation (for the sake of clarity secant lines are not reported in figure).

Figure 10 and Table 4 report results obtained for four-point bending simulations. With reference to the MV_1_ and MV_2_, oscillations are due to nails overlapping and crossing during loading. Also, according to this last loading condition, MV_1_ and MV_2_ have produced a similar behavior, while TP stiffness resulted markedly higher (up to 3 times for *k*_*S*__8400 Nmm_).

**Table 4:** Bending stiffness parameters.

	**MV_**1**_**	**MV_**2**_**	**TP**
**BENDING SIMULATIONS**			
*k*_*T* 900 Nmm_ [Nmm/°]	183.74	172.77	602.36
*k*_*S* 900 Nmm_ [Nmm/°]	122.45	84.99	227.85
*k*_*S* 8400 Nmm_ [Nmm/°]	370.53	440.25	1532.85

## Discussion

Elastic self-locking nails are being used in the medical practice since 90 years, and a few works dedicated to the study of these devices (Tennant et al., 2001; Madan et al., 2003; Martínez et al., 2004; Rose et al., 2013) report their advantages, such as the possibility of avoiding the reaming procedure and the distal inter-locking screws implantation, and a reduced X-ray exposure for patients. At the same time, these same authors cite some causes of concern, mainly related to device stability which have suggested the necessity of performing further studies. Surprisingly, the biomechanical performance of these nails was studied only through clinical studies and some rare mechanical experiments (Blum et al., 2005). Therefore, setting up numerical models for the pre-clinical study of the behavior of these devices is an interesting option since the exact same experimental conditions can be replicated (e.g., same bone properties and morphology).

The first step of this study, reported in a previous work (Pascoletti et al., 2018), was the optimization of the multibody modeling strategies for the simulation of an existing expandable nail design (MV_1_). The second step was the design of two new elastic self-locking nail concepts: an optimized version of the MV_1_ design, obtained through some minor improvement of its morphology (MV_2_), and a completely new device (the TP nail) aimed at achieving a higher stiffness and stability. Following a well-established approach in literature, the stiffness behavior of the implanted fractured bone was considered as a performance index of the nail behavior. A second index related to the device stability, the stability index (SI), was proposed, computable as the sum of contact forces between the nail and the bone.

Behaviors of the three devices are considerably different with reference to tensile loads (Table 2). When a tensile load is applied, there is an initial range of forces where the axial translation of the distal bone fragment is negative (with reference to the x direction), as can be seen in Figure 8A. This effect is due to the curvature of the elastic rods which, during the nail opening, exert a force on the medullary channel moving the distal fragment proximally; once the tensile force overcomes the opposite action of the elastic rods, the distal fragment starts to move distally in the positive x direction. In particular, this occurs for a loading force equal to about 10 N for the MV_1_, 14 N for the MV_2_ and 60 N for the TP; in correspondence of these values the translational displacement becomes positive and these points were used for the evaluation of the tangent stiffness *k*_*T in*_. Specifically, the TP nail is the stiffest one, with an initial slope *k*_*T in*_ of about 22 N/mm; in addition, when the device is implanted, the distal fragment undergoes 50 mm axial displacement only when the applied load reaches its maximum value (100 N), against 23 N and 31 N reported for the other two devices. These results prove that the friction force between the nails and the bone is much more relevant for TP device.

Compression simulations have produced very similar findings for what concerns the *k*_*T in*_ and the *k*_*S*__15 mm_ parameters; on the contrary, with reference to *k*_*T*__0.1 mm_, the TP device exhibits the lowest value. This behavior is due to the fact that the higher friction force which characterizes the TP behavior, does not play any influence on the device resistance when the force is initially applied; on the contrary, it becomes predominant only at a later stage where the TP device is the stiffest one and the system behavior is similar to the one reported for tensile simulations.

Torsion simulations have shown that the TP device exhibits the highest value of *k*_*T*__50 Nmm_ parameter as well as the smallest rotations, these last being lower than 1/10–1/100 those obtained with MV_1_ and MV_2_. The shortening of the MV_1_ elastic rods, leading to the MV_2_ design, produced and increase of torsional stiffness, especially for positive torsion (Table 3). The TP nail showed a more compliant behavior during the negative torsion than the positive one, resulting in lower *k*_*S*__50 Nmm_ and *k*_*S*__100 Nmm_ values.

Bending stiffness of the bone-device system is, once again, higher for the TP device. For this device, bending vs. rotation curves (Figure 10) include a linear tract, beyond 4° of bending rotation; this occurs when the internal wall of the medullary canal comes in contact with the external fixed cylinder and so the stiffening action does not rely any longer on the elastic rods.

All these results are consistent with results concerning the stability index SI which predicted similar performances for the MV_1_ and MV_2_ devices, showing a low influence of the elastic rods geometry on the bone-device interaction and a better performance of the TP device.

According to clinical studies, the improved stiffening action of the TP could widen its applicability to bones undergoing significative loads. In facts, clinical studies concerning the Marchetti-Vicenzi device have produced contrasting results: excellent recovery times and final mobility were reported for humerus diaphyseal fractures (Tennant et al., 2001; Martínez et al., 2004); in contrast, with reference to femoral fractures, the system has not resulted to provide an adequate stability according to some studies (Anastopoulos et al., 2001; Madan et al., 2003), leading to implant failure or long healing periods.

In spite of the many benefits exhibited by the TP nail, one more aspect must be noted. During the whole opening phase, the contact forces of MV_1_ and MV_2_ nails have mainly a normal component, being the friction force negligible; on the contrary contact forces for the TP device include both a normal and a tangential component and the latter is the most important since it can reach 250 N, and this implies that:

1. Elastic rods sliding inside the bone cavity produce an undesirable brushing effect on the medullary canal wall, leading to the risk of damage of the medullary canal itself
2. The surgeon is required to exert a relevant force to implant the device.

According to these considerations, TP device design could be further improved, in order to find a trade-off between its good stiffening performance and its interaction with the bone cavity.

Having excluded the contribute given by the contact forces between the two fractured bone portions is a limitation of this study. However, this approach allowed to produce as general as possible results since different fracture types are likely to behave differently in relation to the applied loads (Zhou et al., 2015). In addition, these numerical experiments are not simulating bone remodeling leading to fracture solidarization and, eventually, to bone-nail adhesion. Moreover, the modeling of the bone as well as some device components as solid bodies could represent a limitation since stress distributions and bone deformations cannot be taken into account. It should be also noted how the position of the distal ends of the rods along the medullary canal plays a substantial influence on the stability of an elastic self-locking nails. In the present study this variability has been canceled for sake of comparison, by positioning the distal ends in the same portion of the medullary canal for all three designs. Undoubtedly, however, a critical condition characterized by a diaphyseal shaft fracture (32-A3 according to the AO classification) has been recreated here, requiring an extremely distal positioning of the rods ends. A fracture located in a region with a smaller canal diameter and a less flared canal surface would allow a further shortening of the rods, thus facilitating the stabilization of the fracture.

As far as the validation setup is concerned, the use of ABS instead of biomechanically realistic materials for the manufacturing of the 3D printed bone fragment, represents a limitation of this study. However, considering the working principle of the expandable nails, it was initially hypothesized that the geometry of the medullary canal might play a primary role in influencing the devices performance. Therefore, in the present study, material properties were overlooked but their contribution will be considered for future developments. Nevertheless, the 3D printing of the femur fragment allowed to obtain, directly in-house, an exact replica of the modified bone geometry included into the performed numerical simulations. Moreover, the addition of customized appendices to the printed model allowed to easily fix the bone fragment on the base of the testing machine without additional and cumbersome locking expedients.

As a matter of fact, the amount of the required stiffness and stability has not been standardized yet. However, this numerical approach can allow making comparisons among different devices and contribute to understand the respective clinical performance, leading to an improved design.

## Conclusions

This work illustrates a numerical comparison between three different intramedullary fixation devices. Starting from the existing Marchetti-Vicenzi nail (MV_1_), an improved version of this design was created shortening the elastic rods and increasing their curvature (MV_2_); the last analyzed devise (TP) is based on a completely new concept, whose design was driven by the previous results obtained on the MV_1_. Experimental tests were performed on the Marchetti-Vicenzi nail and results compared to the MV_1_ model numerical simulations. For the torsional tests, a good correspondence was found between experimental and numerical results, while the tensile test has provided only qualitative results. Tension/compression, positive/negative torsions, and bending loads were simulated for the three models, in order to evaluate the stiffening action of the devices in response to different external loads and to compare devices mechanical behavior. TP nail was proved to provide the best stiffening action for all the applied load, while MV_1_ and MV_2_ showed comparable performances, proving the low impact of the change in the elastic rods geometry. Even though, for TP nail, the device-bone interaction is very good in terms of mechanical stiffness, the behavior of this device is affected by the presence of a high frictional force component of the contact force between the rods and the bone during the opening phase, leading to possible damage to the medullary canal and to complications during the insertion procedure; these results can be used to improve the TP device design. The developed numerical-experimental framework for the virtual testing of innovative elastic self-locking nails has allowed the direct comparison between the different solutions, proving how a numerical approach can be a powerful tool to support the nail design.

## Data Availability Statement

The datasets generated for this study are available on request to the corresponding author.

## Author Contributions

AA and EZ have supervised the full research work. GPu and MT have set up experimental tests. GPu, MT, and GPa have set up and performed numerical simulations. EZ and GPa have analyzed numerical data. EZ and GPa have written the article. All authors have reviewed the article.

## Conflict of Interest

The authors declare that the research was conducted in the absence of any commercial or financial relationships that could be construed as a potential conflict of interest. The reviewer DP declared a past co-authorship with one of the authors MT to the handling Editor.
